# Yellow fever disease: density equalizing mapping and gender analysis of international research output

**DOI:** 10.1186/1756-3305-6-331

**Published:** 2013-11-18

**Authors:** Matthias Bundschuh, David A Groneberg, Doris Klingelhoefer, Alexander Gerber

**Affiliations:** 1 Institute of Occupational, Social and Environmental Medicine, Goethe-University, Theodor-Stern-Kai 7, 60590 Frankfurt am Main, Germany

## Abstract

**Background:**

A number of scientific papers on yellow fever have been published but no broad scientometric analysis on the published research of yellow fever has been reported.

The aim of the article based study was to provide an in-depth evaluation of the yellow fever field using large-scale data analysis and employment of bibliometric indicators of production and quantity.

**Methods:**

Data were retrieved from the Web of Science database (WoS) and analyzed as part of the NewQis platform. Then data were extracted from each file, transferred to databases and visualized as diagrams. Partially by means of density-equalizing mapping makes the findings clear and emphasizes the output of the analysis.

**Results:**

In the study period from 1900 to 2012 a total of 5,053 yellow fever-associated items were published by 79 countries. The United States (USA) having the highest publication rate at 42% (n = 751) followed by far from Brazil (n = 203), France (n = 149) and the United Kingdom (n = 113). The most productive journals are the “Public Health Reports”, the “American Journal of Tropical Medicine and Hygiene” and the “Journal of Virology”. The gender analysis showed an overall steady increase of female authorship from 1950 to 2011. Brazil is the only country of the five most productive countries with a higher proportion of female scientists.

**Conclusions:**

The present data shows an increase in research productivity over the entire study period, in particular an increase of female scientists. Brazil shows a majority of female authors, a fact that is confirmed by other studies.

## Background

The yellow fever disease is an acute viral haemorrhagic disease transmitted by infected mosquitoes [1]. The vast majority of reported cases and deaths occur in tropical areas like Sub-Saharan Africa (about 90%) and to a lesser extent in Central and South America. Yellow fever is endemic in a total of 44 countries, including 32 African countries and 13 Central and South America countries (e.g. Bolivia, Colombia, Ecuador and Peru).

Despite improved prevention options due to the availability of vaccination, yellow fever is still a major public health problem in some countries and of prime importance, since new epidemics have occurred recently. The epidemiology of the yellow fever disease is a dynamic process and dependent on climatic changes such as the occurrence of rainfall or human factors such as migration and travel by plane. Due to the changing global epidemiology of the disease, it is necessary to continuously update the risk areas for yellow fever [2].

A number of recent studies have demonstrated the change of the epidemiology. It is known for example that yellow fever is endemic in many areas of Brazil. Moreno and Barata [3] demonstrated that outbreaks of yellow fever have occurred in regions of Brazil where no reports had been registered for decades [3,4]. According to WHO there are an estimated 200,000 cases of yellow fever causing 30,000 deaths annually worldwide with 90% occurring in Africa. These global estimates of yellow fever disease stem from the 1990s [5-7]. A recent analysis of African data sources due to be published later this year estimates similar figures for 1995, for the year 2013 approximately 84,000 to 170,000 cases and 29,000 to 60,000 deaths of yellow fever [8].

The aim of our study was to perform an in-depth analysis of the quantity and quality of the yellow fever research output worldwide from 1900 to 2012 by using scientometric tools such the citation rate and h-index. We also examined the geographical distribution of items on yellow fever, analyzed the international cooperation and conducted a gender analysis.

## Methods

### Data source

Data were retrieved from the Web of Science (WoS) database by Thomson Reuters. In order to approximate the overall number of published items on yellow fever, data was analyzed regarding the following search term: “yellow fever” OR “fievre jaune” OR “gelbfieber” OR “vomito negro” OR “yellow jack”. In total 5,053 publications were identified and further analyzes were performed using the function “citation report” and the “analyze results” function of the WoS.

The present study is a scientometric analysis of the scientific publications on yellow fever from 1900 to 2012 inclusively. Results from 2013 were not included in the study because the data collection from 2013 is not yet complete.

By using scientometric tools an investigation was performed to assess quality and quantity of research activity for the total number of published items and citations, the average citation per item (citation rate), for single journals, for single countries and for single scientists. The analysis is based on the NewQis platform [9].

### NewQis

The NewQis platform is an international project that uses classical scientometric tools together with new visualisation techniques such as density equalizing calculations to visualize mapping of research activity and quality for benchmarking processes. The project is entitled New Quality and Quantity Indices in Science (NewQis). NewQis can be used to compare countries, institutions or scientists for their academic performance and was established in 2009 [9]. Since then a large number of studies using this platform have been published [10-19].

### H-index

This index is useful to examine the research performance of scientists, countries and institutions. It was developed by the American physicist Jorge Hirsch in 2005. Most of the cited publications and the number of received citations are graded [20,21]).

### Impact factor

The Impact Factor is another scientometric tool for assessing the quality of a journal.

It is calculated from the number of citations of a journal in a given period and the number of articles published in each journal. It can be used to compare the relative importance of a journal in its scientific field [22-24].

### Comparison of countries

Density Equalizing Mapping Projection (DEMP) was described by Gastner and Newman [25] which is the basis of the NewQuis platform established by Groneberg-Kloft *et al*. in 2008 [26]. With this method it is possible to visualize the distribution of the total number of published items and the average citation rate in a country-specific manner. Geographical regions are placed in relation to selected variables (e.g. the number of published items on yellow fever and average citation rate). Specific calculations were based on Gastner and Newman´s algorithms [25], published in 2004. These calculations use a diffusion equation in the Fourier domain borrowed from elementary physics, which allows variable resolution by tracking moving boundaries [25].

### Cooperation analysis

An analysis was performed with regard to the cooperations between institutions, countries and authors. The visual representation of the cooperations was conducted in spider charts with different color intensity.

### Gender analysis

We examined the proportion of male and female researchers listed in the publications of yellow fever, the share of the first and last authorship in the different countries as well as regarding the subject areas. This proved to be problematic, in particular, with the assignment of Asian, especially Chinese names, which are not assignable to a gender but always used for both genders equally. Additionally some author’s Christian names were missing or only indicated as initials. Subsequently, not all author’s genders could be determined. Up to approximately 50 percent of the overall authors could be specified regarding their genders.

Since this research was not carried out on animals or humans, an approval was not needed.

## Results

### Total numbers of published items

**Figure 1:**
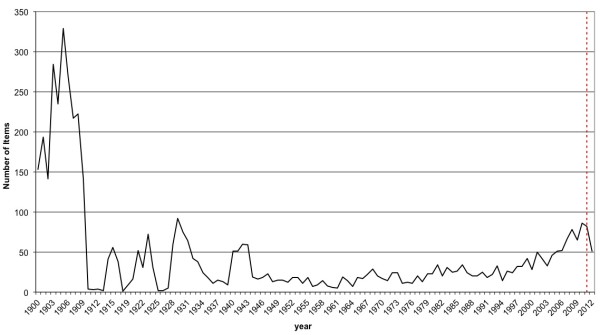
Temporal evolution of the number of publication during the period of the study.

The present study outlines a scientometric analysis of the scientific publications on yellow fever during the research timeframe from 1900 to 2012. In total, 5,053 scientific publications were identified in the WoS database. The analysis revealed an undulating, fluctuating course but in total a continuously increasing process of items until 2010. Most of publications are at the beginning of the 20^th^ century (Figure 1).

**Figure 2:**
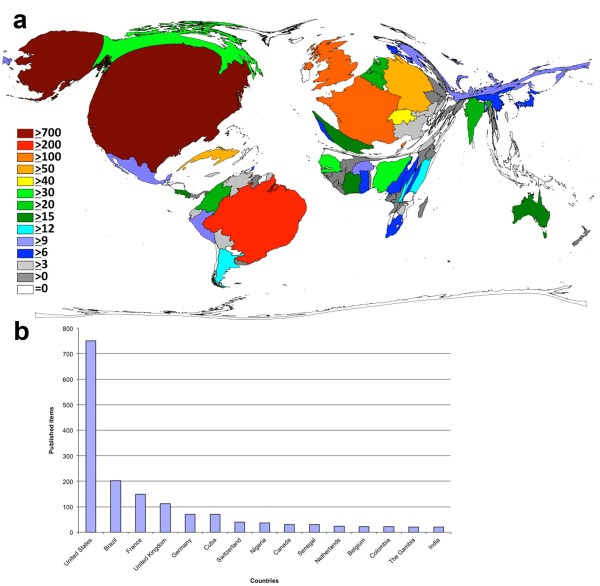
**The most publishing countries relating to the yellow fever disease. a**: DEMP illustrating the number of items in each particular country. The area of each country was scaled in proportion to its total number of publications concerning yellow fever, Legend: Number of published items, Threshold 30 published items. **b**: Ranking of country total number of published.

A country-specific analysis showed the United States as the most productive country, hence dominating the cartogram. The United States have published most of the publications on the yellow fever disease (42% of all publications) followed by Brazil with 11%, France (8%) and the United Kingdom (6%) (Figure 2a and 2b). The countries of the African continent, Asia and the eastern European countries appear reduced and distorted in part on the cartogram (DEMP), the number of publications from these countries is less than 250 items.

**Figure 3:**
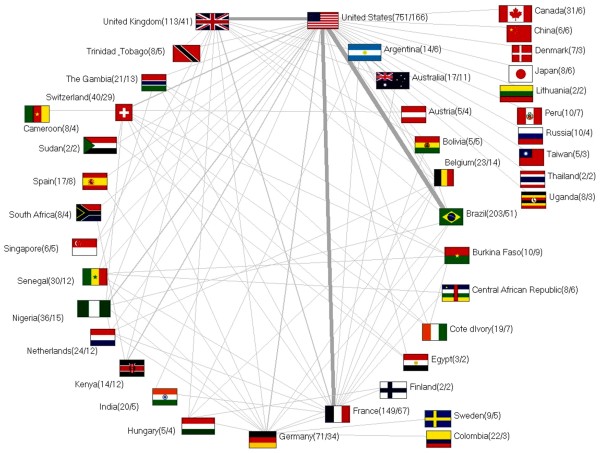
Analysis of international cooperation, number in brackets (published items/number of citations).

The analysis showed also the importance of the United States with regard to international research cooperations, the scientific performance is concentrated in the USA. In total, the United States have 751 international publications, with 166 items they are by far the country which has most frequently collaborated with other countries. Mainly the USA cooperates with South America (Brazil) (n=32 items) as well as the European countries (France and the United Kingdom with 25 items each) (Figure 3).

**Figure 4:**
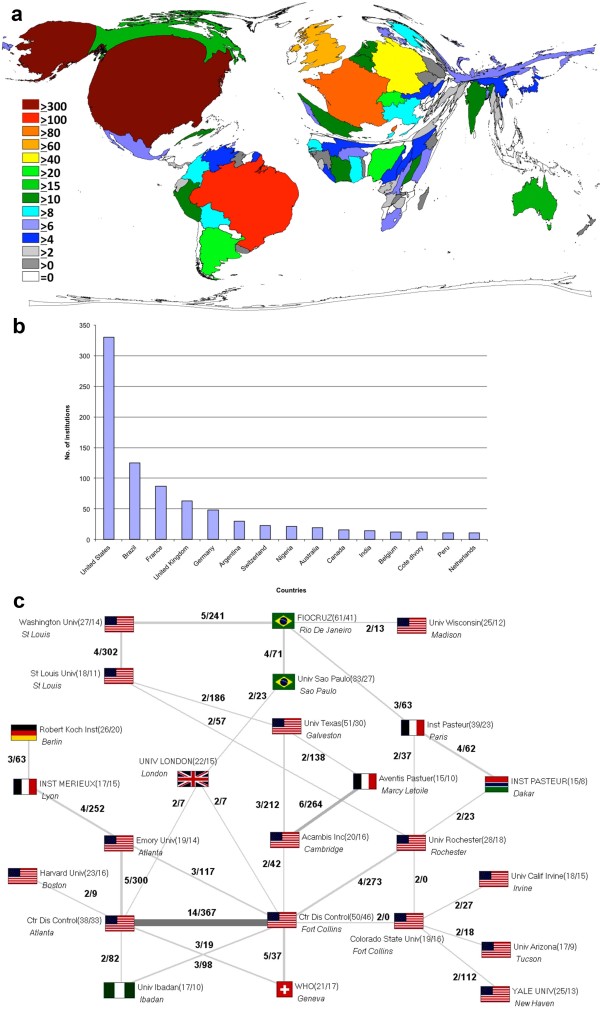
**The most scientific institutions and cooperation concerning yellow fever. a**: DEMP showing the number of scientific institutions, Legend: number of institutions. **b**: Number of national institutions of each country. **c**: Analysis of interinstitutional cooperation, numbers in brackets (published items/number of cooperations), numbers on the beams (cooperations between the two countries/number of citations of these cooperation articles).

Most national research institutions are also in the USA (n=330), followed by Brazil (n=125), France (n=87) and the United Kingdom (n=63) (Figure 4a+b). Most of the inter-institutional cooperation for yellow fever exists between the Centers for Disease and Control Institute in Atlanta and the Centers for Disease and Control Institute in Fort Collins (Figure 4c).

### Citation parameters

**Figure 5:**
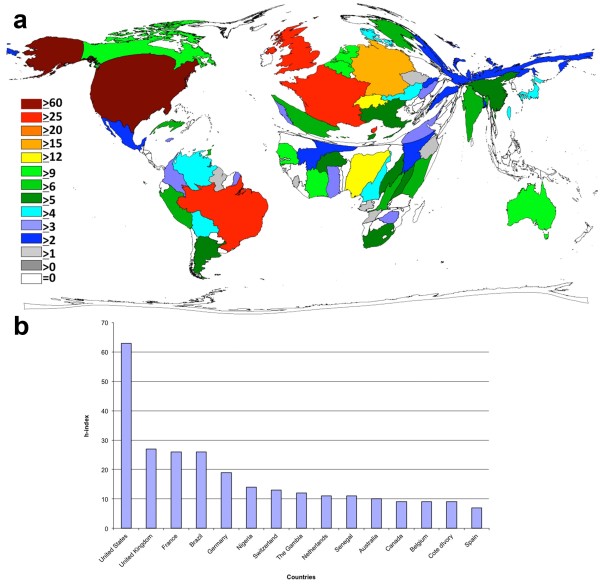
**The modified h-Index of the countries publishing on yellow fever. a**: DEMP showing the modified h-Index of each particular country, Legend: modified h - Index. **b**: Top 15 ranking of countries regarding their modified h-index.

The analysis of the modified h-index for the countries showed that the United States have the highest modified h-index. Thus, the United States is the only country whose h-index is above 60. The USA is recognizably the clearly dominant country in the cartogram (Figure 5a+b) followed by the United Kingdom (27), Brazil and France, which have both an h-index of 26.

### Analysis of the journals

**Figure 6:**
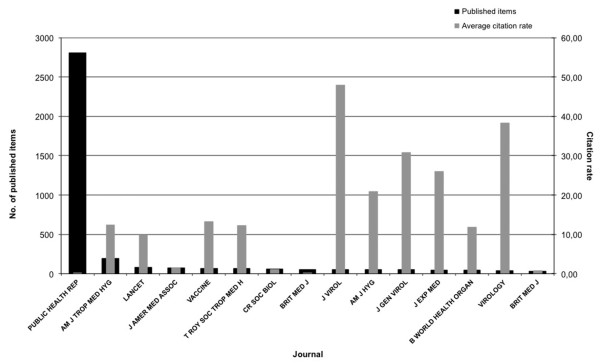
**Top 15 ranking of the most productive journals and their citation rate.***(Public Health Rep: Public Health Reports, Am J Trop Med Hyg: American Journal of Tropical Medicine and Hygiene, Lancet: The Lancet, J Amer Med Assoc: Journal of American Medical Association, Vaccine: Vaccine-Journal, T Roy Soc Trop Med H: Royal Society of Tropical Medicine and Hygiene, CR Soc Biol, Brit Med J: British Medical Journal, J Virol: Journal of Virology, Am J Hyg: American Journal of Hygiene, J Gen Virol: Journal of General Virology, J Exp Med: Journal of Experimental Medicine, B World Health Organ: Bulletin of the World Health Organisation, Virology: Virology - Journal)*.

The vast majority of items regarding yellow fever are being published in the journal *Public Health Reports*. It was identified as the most productive journal with a total of 2,806 items. The citation rate averages < 1. The second place regarding productivity holds the *American Journal of Tropical Medicine and Hygiene* (190 items) with a citation rate of 12.43. But the *Journal of Virology* reached the highest value of citation rate (47.9) with 51 items. Another important journal is the *Virology* (citation rate of 38.34 and 41 items) (Figure 6). The other journals do not differ much in the number of items (30–83 published items).

### Analysis of the authors

**Figure 7:**
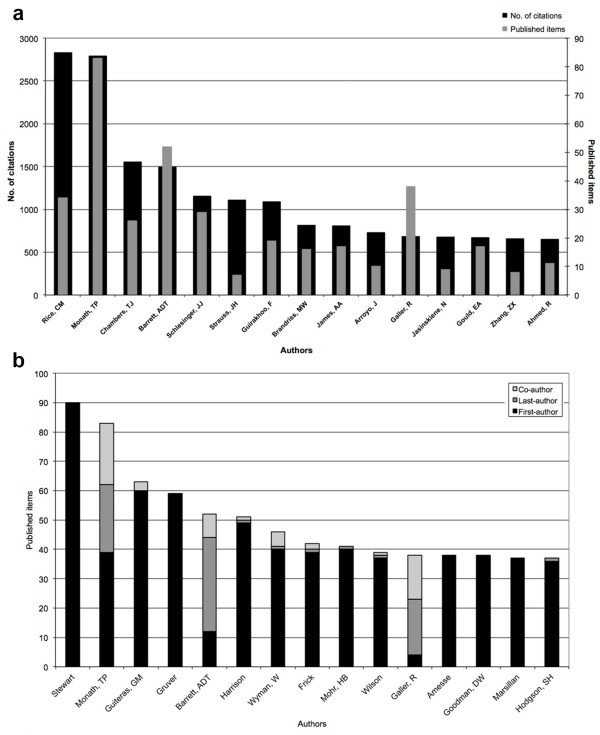
**The most cited authors referred to their published items and their authorship. a**: Top 15 most cited authors and their published items. **b**: 15 most productive authors regarding the proportion of first-authorships and senior - authorship to co-authorship.

The analysis of the 15 most cited authors showed that the scientist *Charles Rice* can claim most citations on his publications. The author is in total 2,830 times cited but has only 34 publications altogether. Almost as often (2,790 times) the author *Thomas Monath* is quoted, but he has more publications (83). He is followed by *Thomas Chambers* and *Alan Barrett*, whose scientific papers are cited 1,552 and 1,495 times from 26 and 52 publications (Figure 7a). In a further analysis we examined the largest qualitative share of the publications. Regarding first- and senior-authorships, *Stewart* has most of the first authorships. He was also the first author in all of his 90 publications. *Stewart* is followed by *Juan Guiteras* and *Gruver. Thomas Monath* has the largest proportion of first, last- and co-authorships of all authors. The scientist *Alan Barrett* has the largest share in last authorship (Figure 7b).

### Analysis of assigned subject areas

**Figure 8:**
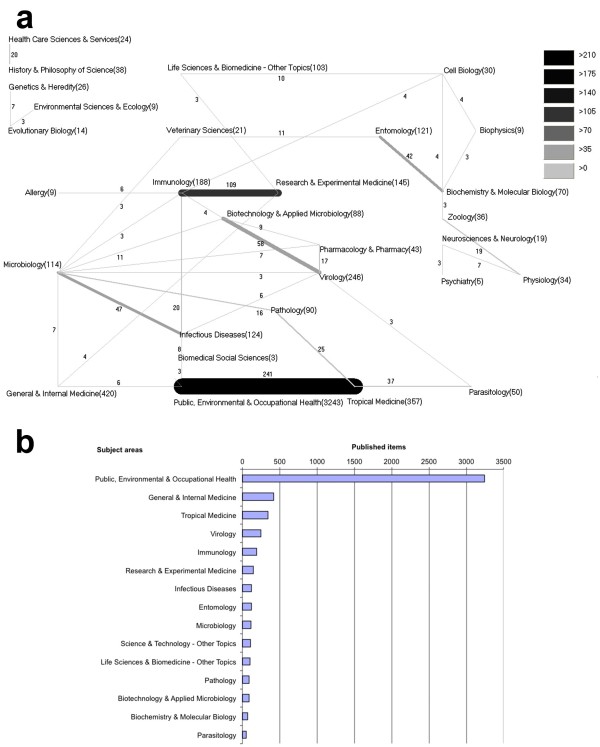
**The most frequent subject areas sorted by combinations and number of items. a**: Most frequently combined subject areas, numbers in brackets (published items), numbers on the beams (combined publications) **b**: The 15 most applied subject areas sorted by number of publications.

The subject area *Public Environmental & Occupational Health* has been published the most (3243 items) in respect to yellow fever. Furthermore, *General & Internal Medicine* (420) and *Tropical Medicine* (357) got second and third place in this ranking (Figure 8b). The analysis found that the categories *Public Environmental & Occupational Health* and *Tropical Medicine* were most frequently combined (241) (Figure 8a).

### Gender analysis

**Figure 9:**
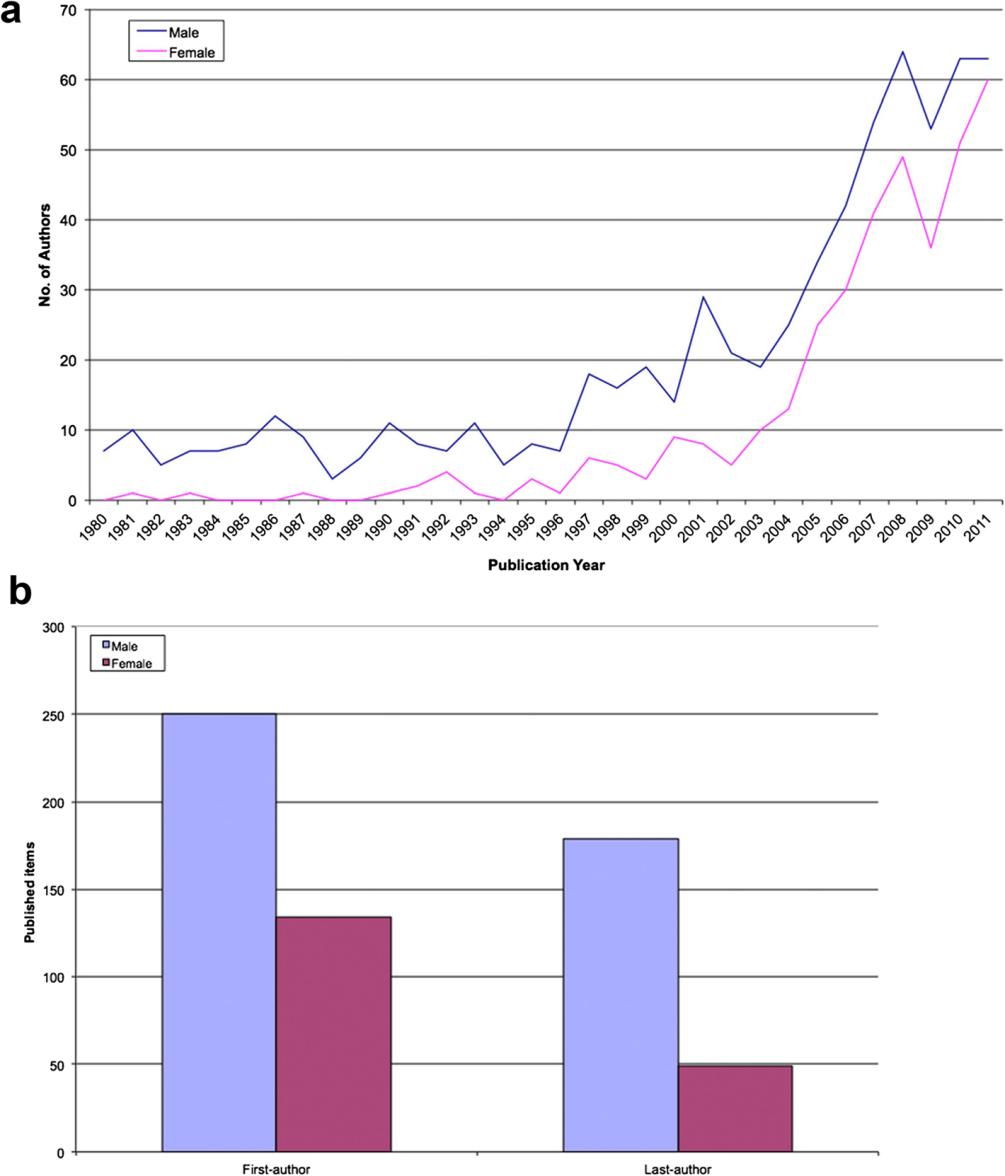
**Gender-analysis of the authors publishing on yellow fever. a**: Gender - analysis of the authors. **b**: Gender - analysis proportion of first - and last authorship.

The analysis regarding the gender distribution of the authors showed that over the entire time period, a total of more male than female scientists were involved in the publications of yellow fever. A steady overall increase of female authorship between 1980 until 2011 was determined. Mainly in the 1990's and the first decade of the 21^st^ century saw a significant increase of female scientists. In 2011 female researchers reached with a number of sixty the highest value within the entire period of study compared to the beginning of the measurement registers. The male authorship also shows an increase from the 1980's to the 20^th^ century. Seven male researchers are registered in 1980, the highest measured value is reached for the whole period of the study in 2008 with 64 male scientists (Figure 9a).

In all publications male scientists have a higher proportion of first and last authorship compared to female authors (Figure 9b).

**Figure 10:**
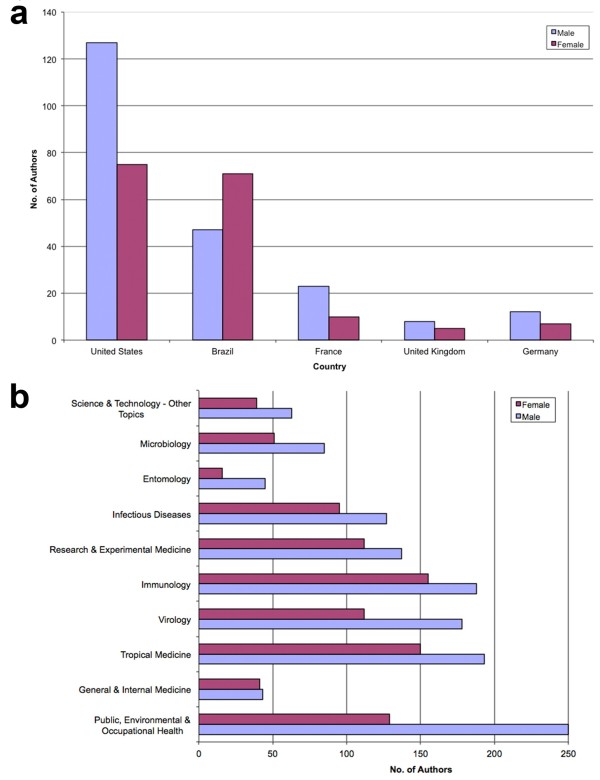
**Gender-analysis of the publishing countries and subject areas. a**: Gender-analysis of the countries. **b**: Gender-analysis of subject areas.

A country-specific gender analysis showed Brazil as the only country out of the five most productive countries with a higher proportion of female scientists. In the other four countries (USA, France, United Kingdom and Germany) there is a higher percentage of male authors (Figure 10a).

The analysis of the gender distribution regarding the 15 most assigned subject areas revealed in all WoS specific categories a higher proportion of male authors compared to female authors. An exception is the category *General & Internal Medicine*, here there are roughly equal numbers of female and male authors (Figure 10b).

## Discussion

Considering the entire time frame of the study, there is an increasing interest in the field of yellow fever, reflected by the highest number of publications already in the early years of the 20^th^ century. However, there is, despite of the overall lower level an slight increase in the amount of publications until 2010. How can we explain this increased scientific interest in the early years of the 20^th^ century, this rapid increase in scientific publications and this development of literature during this period? The answer required a historical review on the investigation of the yellow fever disease. One reason is probably the groundbreaking discoveries of the US-American pathologist and microbiologist Walter Reed. Reed was able to prove that the yellow fever virus is a filterable agent transmissible from mosquito bites [27]. Through his important research results, yellow fever could be brought under control due to the enhanced protection of mosquito bites with mosquito nets. A further increase in publication numbers could be determined in 1929, 1943 and 2010. One explanation for these incidents could be that the yellow fever virus was isolated first in West Africa in 1927 [28], in 1937 the first vaccine was explored by the biologist Max Theiler [29], and in 1943 there was a yellow fever epidemic in Bolivia and in 2008 in Paraguay [30,31]. Furthermore a slight but continuous increase in the number of publications is noted from the 1990's until 2010. New computer technologies and communication via World Wide Web could also be responsible for this increase of publications since 1990.

The analysis of a country-specific manner showed the USA being the country with a leading position in the international publication effort (Figure 2a+b). This may depend on the fact that the USA are equipped with most of the national research institutions (Figure 4a+b). The USA also have a predominant position in the national as well as the international cooperation (Figures 3+4c). Important is also the reputation of a country regarding it’s scientific research. Thus, foreign scientists have a greater interest for carrying out their research in highly esteemed countries as the USA or the United Kingdom because they can conduct their research at likewise prestigious institutions in these countries. Some countries like Columbia, Canada or India collaborate on a relatively low level compared to their number of publications. Other countries like Bolivia, China or Burkina Faso show the opposite results. A high degree of international cooperation usually means a higher number of citations because publications of an international cooperation usually lead to a higher impact compared to items from only one country [24,32,33].

One other bibliometric study concerning a vector transmitted disease (e.g. leishmaniasis, also published in *Parasites & Vectors*) confirmed the USA and Brazil as leading scientific countries on leishmaniasis research [34]. Another bibliometric study concerning malaria also showed the USA as the most productive country [35].

The analysis of the citation rate shows that smaller countries such as Lithuania and Myanmar have high citation rates, it should be considered due to the relatively low number of publications in these countries, that there is the possibility of a bias in the results. Scientometric tools like h-index and citation rate should nevertheless be viewed critically due to the possibility of bias of self-citation and co-authorship.

To compare the quality with the quantity of the publishing journals, the citation rate was examined as a parameter for scientific quality of the publishing journal. The analysis showed that the vast majority of publications regarding yellow fever are being published in the journal *Public Health Reports*, but the *Journal of Virology* reached the highest values of citation rates (Figure 6). Other important journals regarding the area of yellow fever are the *American Journal of Tropical Medicine and Hygiene* and the *Virology. Public Health Reports* is the official journal of the U.S. Public Health Service. This journal already published in 1878 for the first time. It provides informative data for practically employed doctors, professors and students of public health. The peer-reviewed journal publishes findings of the current scientific research, leading important discussions about important issues of public health community. In editions of the *Public Health Reports* from the early 20^th^ century, there are reports and statistics on the morbidity and deaths due to the yellow fever disease. The *Public Health Reports* are published by the *Association of Schools of Public Health* since 1999 [36].

Regarding the origin of the 15 most cited authors, the analysis showed that 9 of 15 authors are of U.S. American origin. This fact is probably related to the high number of U.S. institutions and the high number of collaborations between U.S. institutions. The U.S. American scientist *Thomas Monath* has the largest proportion of first, last- and coauthorship, *Stewart* (USA), however, is the author with most first authorships. The scientist *Alan Barrett* has the largest share of last authorships (Figure 7b).

The analysis of the subject areas according to the original WoS categories found that the categories *Public Environmental and Occupational Health* and *Tropical Medicine* were most frequently combined, in these two categories the most common published items for yellow fever disease could be found. Other important subject areas are *Immunology* and *Experimental Medicine*. It is likely that these areas of medicine dealing especially with the pathogenesis of the disease and especially the exploration of a curative treatment option for yellow fever, which is the focus of further ongoing research.

Most published items on yellow fever can be found in the WoS category *Public, Environmental & Occupational Health*, probably because of the high proportion of publications in the journal *Public Health Reports* in the first years of the analyzed time period (Figure 8b).

The gender analysis showed a steady increase of female authorships between 1980 until 2011. Mainly in the 1990's and the first decade of the 21th century saw a significant increase of female scientists. This study could show that Brazil is the only country with a higher proportion of female scientists. This is confirmed by a study of the *Konrad-Adenauer Foundation* in Germany which found that women despite a lot of disadvantages are in the majority at the universities in Brazil [37]. A study of numerous gender benchmarking studies concerning Brazil of Women in Global Science & Technology (Wisat) and the Organization for Women in Science for the Developing World (OWSD) was able to confirm the leadership of women in Brazil in science and research [38].

## Conclusion

Up to date there are numerous reviews about yellow fever in the literature, but no in-depth scientometric analysis. The purpose of this study was to accomplish the first scientometric analysis and visualization of research output on yellow fever including a detailed investigation of gender aspects. By using scientometric tools and the method of DEMPs it was possible to assess the scientific quantity and quality of the publications.

The analysis showed that the male researchers are principally in a majority. Only Brazil showed a higher number of female authors.

The United States leads the international scientific output on yellow fever disease followed by Brazil, France, the United Kingdom and Germany. The USA holds the highest h-index and most of the productive authors and institutions.

## Competing interest

The authors declare that they have no conflict of interest.

## Authors’ contributions

MB, DG, DK and AG have made substantial contributions to the conception and design of the study, acquisition of the data and interpretation. They have been involved in drafting and revising the manuscript. All authors have read and approved the final manuscript.

## References

[B1] Thomas RE Lorenzetti DL Spragins W Jackson D Williamson T Active and passive surveillance of yellow fever vaccine 17D or 17DD-associated serious adverse events: Systematic review Vaccine 2011 29 4544 4555 10.1016/j.vaccine.2011.04.055 21549787

[B2] Jentes ES The revised global yellow fever risk map and recommendations for vaccination, 2010: consensus of the Informal WHO Working Group on Geographic Risk for Yellow Fever (vol 11, pg 622, 2011) Lancet Infect Dis 2012 12 98 98 10.1016/S1473-3099(11)70147-5 21798462

[B3] Moreno ES Barata RDB Methodology for Definition of Yellow Fever Priority Areas, Based on Environmental Variables and Multiple Correspondence Analyses Plos Neglect Trop D 2012 6 7 1 7 10.1371/journal.pntd.0001658 PMC3389021 22802971

[B4] Wamala JF Malimbo M Okot CL Atai-Omoruto AD Tenywa E Miller JR Balinandi S Shoemaker T Oyoo C Omony EO Epidemiological and laboratory characterization of a yellow fever outbreak in northern Uganda, October 2010–January 2011 Int J Infect Dis 2012 16 e536 e542 10.1016/j.ijid.2012.03.004 22575876

[B5] Heinz FX Stiasny K Flaviviruses and flavivirus vaccines Vaccine 2012 30 4301 4306 10.1016/j.vaccine.2011.09.114 22682286

[B6] Quaresma JAS Pagliari C Medeiros DBA Duarte MIS Vasconcelos PFC Immunity and immune response, pathology and pathologic changes: progress and challenges in the immunopathology of yellow fever Rev Med Virol 2013 23 305 318 10.1002/rmv.1752 23873723

[B7] Séralini G-E Clair E Mesnage R Gress S Defarge N Malatesta M Hennequin D de Vendômois JS Long term toxicity of a Roundup herbicide and a Roundup-tolerant genetically modified maize Food Chem Toxicol 2012 50 4221 4231 10.1016/j.fct.2012.08.005 22999595

[B8] Garske T Yellow fever burden estimation: Summary. Manuscript in preparation, summary of methods/findings 2013 [http://www.who.int/csr/disease/yellowfev/YellowFeverBurdenEstimation_Summary2013.pdf]

[B9] Groneberg-Kloft B Fischer TC Quarcoo D Scutaru C New quality and quantity indices in science (NewQIS): the study protocol of an international project Jom- J Occup Med (London, England) 2009 4 16 10.1186/1745-6673-4-16 PMC2708171 19555514

[B10] Groneberg DA Schilling U Scutaru C Uibel S Zitnik S Mueller D Klingelhoefer D Kloft B Drowning - a scientometric analysis and data acquisition of a constant global problem employing density equalizing mapping and scientometric benchmarking procedures Int J Health Geogr 2011 55 1 10 10.1186/1476-072X-10-55 PMC3229455 21999813

[B11] Groneberg-Kloft B Klingelhoefer D Zitnik SE Scutaru C Traffic medicine-related research: a scientometric analysis Bmc Public Health 2013 13 541 10.1186/1471-2458-13-541 23734726 PMC3681580

[B12] Scutaru C Quarcoo D Takemura M Welte T Fischer TC Groneberg-Kloft B Density-Equalizing Mapping and Scientometric Benchmarking in industrial health Ind Health 2010 48 197 203 10.2486/indhealth.48.197 20424350

[B13] Gerber A Groneberg DA Klingelhofer D Bundschuh M Gout: a critical analysis of scientific development Rheumatol Int 2013 33 2743 2750 10.1007/s00296-013-2805-1 23797780

[B14] Fricke R Uibel S Klingelhoefer D Groneberg DA Influenza: a scientometric and density-equalizing analysis BMC Infect Dis 2013 13 454 10.1186/1471-2334-13-454 24079616 PMC3851602

[B15] Vogelzang BH Scutaru C Mache S Vitzthum K Kusma B Schulte-Herbruggen O Groneberg DA Quarcoo D A bibliometric analysis of bipolar affective disorders using density-equalizing mapping and output benchmarking Indian J Psychiatry 2012 54 320 326 10.4103/0019-5545.104807 23372233 PMC3554962

[B16] Vitzthum K Scutaru C Musial-Bright L Quarcoo D Welte T Spallek M Groneberg-Kloft B Scientometric analysis and combined density-equalizing mapping of environmental tobacco smoke (ETS) research PloS one 2010 5 e11254 10.1371/journal.pone.0011254 20582305 PMC2889821

[B17] Groneberg-Kloft B Quarcoo D Scutaru C Quality and quantity indices in science: use of visualization tools EMBO Rep 2009 10 800 803 19648952 10.1038/embor.2009.162 PMC2726685

[B18] Groneberg-Kloft B Dinh QT Scutaru C Welte T Fischer A Chung KF Quarcoo D Cough as a symptom and a disease entity: scientometric analysis and density-equalizing calculations J Investig Allergol Clin Immunol 2009 19 266 275 19639722

[B19] Kusma B Scutaru C Quarcoo D Welte T Fischer TC Groneberg-Kloft B Tobacco control: visualisation of research activity using density-equalizing mapping and scientometric benchmarking procedures Int J Environ Res Public Health 2009 6 1856 1869 10.3390/ijerph6061856 19578464 PMC2705221

[B20] Hirsch JE Does the h index have predictive power? Proc Natl Acad Sci USA 2007 104 19193 19198 10.1073/pnas.0707962104 18040045 PMC2148266

[B21] Hirsch JE An index to quantify an individual’s scientific research output Proc Natl Acad Sci U S A 2005 102 16569 16572 10.1073/pnas.0507655102 16275915 PMC1283832

[B22] Garfield E The impact factor and using it correctly Unfallchirurg 1998 101 413 414 9677838

[B23] Garfield E The history and meaning of the journal impact factor Jama-J Am Med Assoc 2006 295 90 93 10.1001/jama.295.1.90 16391221

[B24] Zarate V Cerda J Strengths and weaknesses of the impact factor of scientific journals Rev Med Chile 2007 135 1474 1478 18259661

[B25] Gastner MTMTNMEJ Diffusion-based method for producing density-equalizing maps Proc Natl Acad Sci U S A 2004 101 7499 7504 10.1073/pnas.0400280101 15136719 PMC419634

[B26] Groneberg-Kloft B Scutaru C Kreiter C Kolzow S Fischer A Quarcoo D Institutional operating figures in basic and applied sciences: scientometric analysis of quantitative output benchmarking Health res policy and systems / BioMed Central 2008 6 6 10.1186/1478-4505-6-6 PMC2459159 18554379

[B27] Reed W Carroll J Agramonte A The etiology of yellow fever: an additional note. 1901 Mil Med 2001 166 44 53 11569390

[B28] Pisano MR Nicoli J Tolou H Homogeneity of yellow fever virus strains isolated during an epidemic and a post-epidemic period in West Africa Virus Genes 1997 14 225 234 10.1023/A:1007987911220 9311567

[B29] Mortimer P The use of yellow fever virus modified by in vitro cultivation for human immunization (Reprinted from J Exp Med vol 65, pg 787–800, 1937) Rev Med Virol 2000 10 3 13 10.1002/(SICI)1099-1654(200001/02)10:1<3::AID-RMV261>3.0.CO;2-O 10654001

[B30] Bevier G Torresmunoz N Doriamedina J Yellow fever in Bolivia, its history and epidemiology Am J Trop Med Hyg 1953 2 464 482 13040681 10.4269/ajtmh.1953.2.464

[B31] Outbreak news. Yellow fever, Paraguay Wkly Epidemiol Rec 2008 83 105 18350685

[B32] Mohebbi MR The impact of "Impact Factor" on medical journalism in the developing world Indian Pediatr 2008 45 604 604 18695286

[B33] Andersen J Belmont J Cho CT Journal impact factor in the era of expanding literature J Microbiol Immunol 2006 39 436 443 17164944

[B34] Ramos JM Gonzalez-Alcaide G Bolanos-Pizarro M Bibliometric analysis of leishmaniasis research in Medline (1945–2010) Parasite Vector 2013 6 55 10.1186/1756-3305-6-55 PMC3602049 23497410

[B35] Van Eijk AM Hill J Povall S Reynolds A Wong HL Ter Kuile FO The Malaria in Pregnancy Library: a bibliometric review Malaria J 2012 11 362 10.1186/1475-2875-11-362 PMC3522037 23110589

[B36] ASPH Public Health Rep [http://www.asph.org/document.cfm?page=713]

[B37] Konrad-Adenauer-Stiftung Frauen in Brasilien http://www.kas.de/wf/doc/kas_17800-1522-1-30.pdf?091024002708

[B38] Huyer S Hafkin N Scorecard on Gender Equality in the Knowledge Society [http://elsevierconnect.com/brazilian-women-lead-in-science-technology-and-innovation-study-shows/]

